# Scarless Gene Tagging with One-Step Transformation and Two-Step Selection in *Saccharomyces cerevisiae* and *Schizosaccharomyces pombe*

**DOI:** 10.1371/journal.pone.0163950

**Published:** 2016-10-13

**Authors:** Dirk Landgraf, Dann Huh, Erinc Hallacli, Susan Lindquist

**Affiliations:** 1 Whitehead Institute for Biomedical Research, Cambridge, Massachusetts, United States of America; 2 Department of Molecular and Cellular Biology, Harvard University, Cambridge, Massachusetts, United States of America; 3 Howard Hughes Medical Institute, Department of Biology, Massachusetts Institute of Technology, Cambridge, Massachusetts, United States of America; Tulane University Health Sciences Center, UNITED STATES

## Abstract

Gene tagging with fluorescent proteins is commonly applied to investigate the localization and dynamics of proteins in their cellular environment. Ideally, a fluorescent tag is genetically inserted at the endogenous locus at the N- or C- terminus of the gene of interest without disrupting regulatory sequences including the 5’ and 3’ untranslated region (UTR) and without introducing any extraneous unwanted “scar” sequences, which may create unpredictable transcriptional or translational effects. We present a reliable, low-cost, and highly efficient method for the construction of such scarless C-terminal and N-terminal fusions with fluorescent proteins in yeast. The method relies on sequential positive and negative selection and uses an integration cassette with long flanking regions, which is assembled by two-step PCR, to increase the homologous recombination frequency. The method also enables scarless tagging of essential genes with no need for a complementing plasmid. To further ease high-throughput strain construction, we have computationally automated design of the primers, applied the primer design code to all open reading frames (ORFs) of the budding yeast *Saccharomyces cerevisiae* (*S*. *cerevisiae*) and the fission yeast *Schizosaccharomyces pombe* (*S*. *pombe*), and provide here the computed sequences. To illustrate the scarless N- and C-terminal gene tagging methods in *S*. *cerevisiae*, we tagged various genes including the E3 ubiquitin ligase *RSP5*, the proteasome subunit *PRE1*, and the eleven Rab GTPases with yeast codon-optimized mNeonGreen or mCherry; several of these represent essential genes. We also implemented the scarless C-terminal gene tagging method in the distantly related organism *S*. *pombe* using kanMX6 and HSV1tk as positive and negative selection markers, respectively, as well as *ura4*. The scarless gene tagging methods presented here are widely applicable to visualize and investigate the functional roles of proteins in living cells.

## Introduction

The budding yeast *Saccharomyces cerevisiae* (*S*. *cerevisiae*) and the fission yeast *Schizosaccharomyces pombe* (*S*. *pombe*) have both been extensively studied as model systems for eukaryotic cells. These yeast cells are ideal model organisms for functional genomics and genome-wide gene engineering, partly because of their efficient homologous recombination machinery. Budding yeast has about 7,500 predicted open reading frames (ORFs) that each encode a hypothetical protein of >100 amino acids [1], although the total number of actual protein-coding sequences is substantially smaller and still under debate [2]. As of February 2016, the *S*. *cerevisiae* Genome Database (SGD) lists 5,139 verified ORFs, 677 uncharacterized ORFs, and 784 dubious ORFs (see http://www.yeastgenome.org/genomesnapshot). *S*. *pombe* has been reported to contain 5125 protein-coding sequences, of which 76 are dubious (see http://www.pombase.org/status/statistics).

Owing to efficient genetic manipulation, several genome-wide protein fusion libraries have previously been constructed for both yeasts. In *S*. *cerevisiae*, libraries were created by C-terminally tagging 4,500–6,000 yeast ORFs at their endogenous gene loci with, for example, an epitope tag [3] or a fluorescent protein [4,5]. In *S*. *pombe*, large-scale protein localization studies were performed both by using 4910 ORF-YFP fusions integrated at the ectopic *leu1* locus and driven by the inducible *nmt1* promoter [6] or using strains with GFP endogenously fused to 1058 ORFs [7]. The fusion tag is usually added to the C-terminus of the coding region of the gene of interest using a linear DNA fragment that is generated by PCR and integrated into the chromosome with a selection marker [8]. A large number of plasmids with various combinations of tags and selection markers have been developed and can be used as templates for the PCR amplification of the integration cassette [9]. One problem is that most of the C-terminal integration methods, beside a few exceptions [5,10,11], use an exogenous 3’ UTR, which disrupts any endogenous 3’ end mediated expression control of the tagged gene and can hence alter protein levels [12]. Sequences in the 3’ UTR can also control the endogenous sub-cellular localization of the mRNA, which is often coupled to the localization and expression of the protein [13–16]. In addition, it was recently shown that the widely used *CYC1* and *ADH1* 3’ UTRs contain cryptic promoters that lead to abundant convergent antisense transcription, which may also interfere with normal protein expression [17]. Any changes to the UTRs that may affect protein abundances are of concern, especially for quantitative studies that measure protein levels [3,18]. These problems call for gene tagging methods that do not replace the endogenous UTRs or leave behind any unwanted DNA sequences in the tagged gene (i.e. a “scar”).

**Fig 1:**
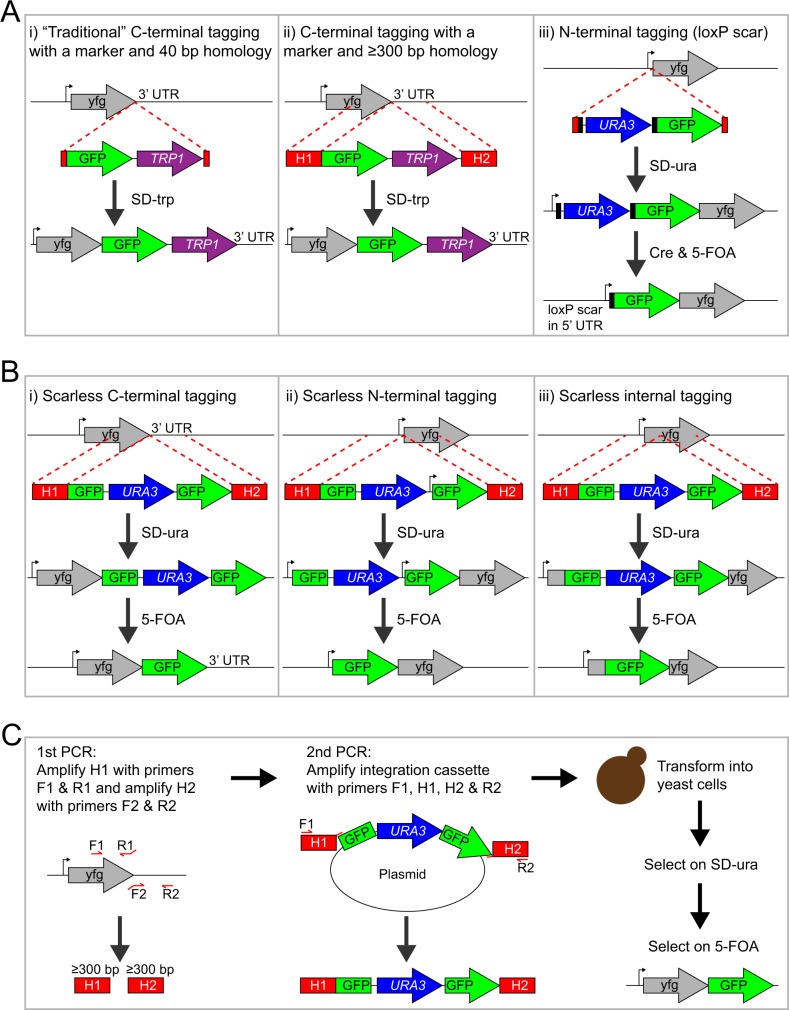
Cartoon of gene tagging methods in yeast. (A) Commonly used methods for tagging a gene of interest (yfg = your favorite gene): C-terminal gene tagging using a marker and 40–50 bp homology (Fig 1A-i) or ≥300 bp homology (Fig 1A–ii), and N-terminal gene tagging using the Cre-loxP system (Fig 1A-iii). Methods i and ii use a selection marker that disrupts the 3’ UTR and cannot be eliminated, which might perturb the function of the fusion. For method iii, the loxP-flanked selection marker can be excised with the Cre recombinase, which leaves behind one flippase recognition target (FRT) site “scar” in the 5’ UTR of the tagged ORF. (B) Tagging methods introduced in this study: scarless C-terminal gene tagging (Fig 1B-i), scarless N-terminal gene tagging (Fig 1B-ii), and scarless internal gene tagging (Fig 1B-iii). The scarless tagging methods require a second round of selection to eliminate the *URA3* marker, which is surrounded by identical GFP sequences. Note that the resulting “GFP scar” becomes a genuine part of the full-length GFP fusion protein after recombination and that the endogenous UTRs are not altered. The scarless N-terminal tagging method can be used for tagging essential genes in haploid yeast cells because a constitutive promoter situated between the *URA3* marker and the second GFP fragment drives expression of the ‘partial GFP’-tagged gene prior to excision of *URA3*. Integration cassettes with either a partial GFP tag (as shown here) or a full-length GFP tag (S1 Fig) can be used. (C) Detailed description of the steps for scarless C-terminal tagging of a gene of interest with GFP. The integration cassette is built using two-step PCR synthesis. The homology arms H1 and H2 are amplified in the first round of PCRs and then used as primers together with F1 and R2 in the second round of PCRs. The primer-binding sites of H1 and H2 are unique, which is important for the efficient PCR amplification of the integration cassettes. Excision of the *URA3* marker is not shown in this cartoon but is identical to the depiction above (Fig 1B-i).

Scarless integration becomes even more important in the construction of an N-terminal fusion at an endogenous gene locus. To allow for gene transcription, N-terminal fusions require excision of the selection marker after the integration or placement of the marker upstream of the promoter. N-terminal tagging of essential genes provides an even greater challenge since expression of the essential gene or, alternatively, expression of a second unmodified gene copy (e.g. from a complementing plasmid) must be ensured during the strain construction process. A number of methods exist for endogenous N-terminal tagging: the Cre-loxP method [19], a 3xHA tag-*URA3*-3xHA tag cassette using positive and negative selection [20], a sequential two-step integration method using positive and negative selection [21], and the I-SceI endonuclease-driven recombination method [10]. All N-terminal tagging methods could theoretically also be extended to the construction of internal protein fusions as demonstrated with the Cre-loxP method and the 9xMyc tag [19]. The main disadvantage of the Cre-loxP method is that excision of the selection marker by the Cre recombinase leaves behind a loxP “scar” (Fig 1A) that might perturb the function or expression level of the tagged protein.

N-terminal tagging is important for proteins, whose function requires a normal, unmodified C-terminus. The eleven Rab GTPases of *S*. *cerevisiae* are good examples. These proteins are post-translationally modified with the addition of prenyl anchors at two C-terminal cysteine residues and these anchors are required for the association of the Rab proteins with intracellular membranes [22]. When tagged C-terminally with GFP, nine *S*. *cerevisiae* Rab proteins show a false, cytoplasmic localization pattern and the two essential Rab proteins, Sec4 and Ypt1, are missing from the C-terminal GFP-tagged ORF library [4]. Plasmid-borne N-terminal fluorescent protein fusions of the Rab proteins Sec4, Ypt1, Ypt6, Ypt7, Ypt31, Ypt52, and Vps21 display distinct subcellular localization patterns [22,23]. However the yeast Rab proteins have not been tagged N-terminally at their endogenous loci, which would avoid potential over-expression artifacts or cell-to-cell variability issues that arise from plasmid copy number fluctuations. Similar problems are expected for many other proteins that do not tolerate a C-terminal tag such as the essential E3 ubiquitin ligase Rsp5 (NEDD4 homolog) [24].

Another important aspect of gene tagging is the transformation efficiency of the cells and the identification of a correct clone. Using an integration cassette with several hundred base pair-long homology arms increases the transformation efficiency by 30 to 50-fold [25,26], which would be advantageous for routine and high-throughput strain construction. The length of the homology arms for gene tagging is usually 40–50 bp for budding yeast and over 80 bp for fission yeast. Such lengths are sufficient for chromosomal integration [26–28]. However, a low transformation efficiency and a substantial amount of off-target integrations are often observed in practice, thus requiring PCR screening of many clones. In addition, short homology overhangs are also more susceptible to sequence polymorphism, which provides a challenge for gene tagging in unsequenced yeast strains and sequence-diverse wild strains. For the more efficient integration method, the several hundred base pair long flanking regions can be generated either by PCR or chemical DNA synthesis. For PCR, four primers in total are required to make the two homology arms that are needed to tag a gene. However, designing quartets of primers for each gene is a repetitive and time-consuming task when done manually. A web interface for automated primer design for tagging *S*. *cerevisiae* genes has been developed [29] but this tool is limited to the traditional two-primer method with short, 40-bp overhangs, which suffers from the low transformation efficiency.

To overcome the limitations described above, we herein present an improved, simpler, more efficient, and inexpensive method for tagging endogenous genes with fluorescent proteins. We applied this method in *S*. *cerevisiae* for the construction of scarless N-terminal and scarless C-terminal fusions at endogenous gene loci using a one-step transformation followed by two-step selection (Fig 1B and 1C). A similar method was applied to the construction of scarless C-terminal fusions in the fission yeast *S*. *pombe*. Our scarless gene tagging methods leave all the strains auxotrophic and chemical markers available for downstream applications. The primer design for the different gene tagging methods (i.e. scarless C-terminal, and scarless N-terminal, and C-terminal with marker) was computationally automated, and was employed to design primers for essentially all *S*. *cerevisiae* and *S*. *pombe* genes. We have deposited with Addgene gene tagging plasmids with the yeast codon-optimized fluorescent proteins mNeonGreen [30], monomeric superfolder GFP (msfGFP) [31], and mCherry [32] for *S*. *cerevisiae*, and scarless C-terminal gene tagging plasmids with mGFPmut3 and the *ura4* marker or the herpes simplex virus type 1 thymidine kinase (HSV1tk) marker for *S*. *pombe*. Thus our methods should be easily utilized and widely applicable.

## Materials and Methods

### Yeast Strains and Growth Conditions

The *S*. *cerevisiae* strains used in this study were derived from W303 and are listed in S1 Table. For *S*. *cerevisiae*, the standard lithium acetate-polyethylene glycol (PEG) transformation protocol [33] was used for chromosomal integration of the gene tagging constructs. The budding yeast strains were usually grown in YPD medium, or synthetic complete (SC) with 2% (v/v) glucose, or a synthetic dropout medium if selection was required.

The *S*. *pombe* strains were derived from strains FWP1 and FWP172, which were gifts from Dr. Fred Winston (Harvard Medical School). The *S*. *pombe* strains are listed in S1 Table. For the transformation of the fission yeasts, the standard lithium acetate method was used [34]. The fission yeast strains were grown in YES medium, unless otherwise noted.

### Plasmid Construction

Plasmids used in this study are listed in S2 Table. The primers that were used for the plasmid construction are listed in S3 Table. *E*. *coli* strain DH5α was used for plasmid construction and plasmid propagation, unless otherwise noted. All cloning was performed with the isothermal assembly method [35], unless otherwise stated, and validated by DNA sequencing.

The sequences of the yeast codon-optimized fluorescent proteins mNeonGreen, msfGFP, and mCherry were created with JCat [36] and synthesized (Genewiz and Integrated DNA Technologies).

Plasmid pDML20 was built by PCR amplifying a linker (5xGA) followed by yeast codon-optimized msfGFP from pUC57-msfGFP (Genewiz) with primers DML_P110_F and DML_P111_R. The PCR product was inserted into pFA6a-GFP (S65T)-kanMX6 [37], which was cut with restriction enzymes PacI and AscI.

Plasmid pDML61 was built by PCR amplifying the *Candida glabrata HIS3* (*C*.*g*. *HIS3*) marker from pNH603 using primers DML_P285_F and DML_P286_R. The PCR product was then cloned into pDML20, which was digested with BglII and PmeI.

Plasmid pDML63 was built by PCR amplifying a linker (5xGA) followed by non-yeast codon-optimized mNeonGreen from pUC57-mNeonGreen (Genewiz) with primers DML_P218_F and DML_P219_R. The PCR product was then inserted into pDML61, which was cut with restriction enzymes SgrAI and AscI.

A DNA fragment containing a linker (5xGA) and yeast codon-optimized mNeonGreen was synthesized (gBlock, IDT DNA), digested with PacI and AscI, and ligated with the instant sticky-end ligase mix (NEB) into pDML61, which was cut with the same restriction enzymes. The resulting plasmid was named pDML99.

Plasmid pDML112 was built by ligating PacI/AscI-digested yeast codon-optimized mCherry (gBlock, IDT DNA) into pA06, which was cut with the same enzymes. Ligation was performed with the instant sticky-end ligase mix (NEB). Plasmid pA06 was a gift from Rishi Jajoo (Springer lab, Harvard Medical School).

Plasmid pDML145 was built by inserting two PCR products into pNH604, which was digested with XhoI and NotI. The first PCR product corresponds to the *TDH3* promoter, which was PCR amplified from genomic DNA using primers DML_P371_F and DML_P405_R. The second PCR product corresponds to yeast codon-optimized mNeonGreen, which was amplified from pDML99 with primers DML_P444_F and DML_P442_R.

Plasmid pDML152 was built by inserting two PCR products into vector pFA6a-GFP (S65T)-kanMX6 [37], which was cut with restriction enzymes PacI and PmeI. The first PCR product corresponds to a linker (5xGA) followed by yeast codon-optimized mNeonGreen, which was PCR amplified from pDML99 with primers DML_P110_F and DML_P461_R. The second PCR product corresponds to the *Candida glabrata TRP1* (*C*.*g*. *TRP1*) marker, which was amplified from pNH604 with primers DML_P462_F and DML_P463_R.

Plasmid pDML166 was built by PCR amplifying the *Saccharomyces paradoxus* (*S*. *paradoxus*) EF1α promoter from a synthetic DNA fragment (gBlock, ITD DNA) with primers DML_P499_F and DML_P500_R. The resulting PCR product was then inserted into pDML145, which was cut with XhoI and ClaI.

Plasmid pDML190 was built in two steps. First, four PCR products were inserted into plasmid pDML152, which was cut with BamHI and SpeI. The first PCR product corresponds to mNeonGreen (amino acid #1–177), which was PCR amplified from pDML166 with primers DML_P555_F and DML_P556_R. The second PCR product corresponds to the *Kluyveromyces lactis URA3* (*K*.*l*. *URA3*) marker, which was amplified from pUG72 with primers DML_P557_F and DML_P558_R. The third PCR product corresponds to the *S*. *paradoxus* EF1α promoter (P_EF1α_), which was amplified from pDML166 with primers DML_P559_F and DML_P560_R. The fourth product corresponds to mNeonGreen (amino acids #60–236) followed by a linker (5xGA), which was amplified from pDML166 with primers DML_P561_F and DML_P562_R. The resulting plasmid lacked a methionine (ATG) and a Kozak sequence downstream of P_EF1α_. Next, we introduced the start codon and Kozak sequence into the plasmid by PCR amplifying another fragment from pDML166 with primers DML_P608_F and DML_P609_R and inserting it into the ClaI/BlpI-digested vector.

Plasmid pDML193 was built by inserting two PCR products and the *K*.*l*. *URA3* cassette into pDML190, which was digested with BamHI and SpeI. The first PCR product corresponds to mCherry (#1–177), which was amplified from pDML112 with primers DML_P619_F and DML_P620_R. The second PCR product corresponds to mCherry (#60–236) followed by a linker (5xGA), which was also amplified from pDML112 using primers DML_P621_F and DML_P622_R. The DNA fragment containing the *K*.*l*. *URA3* marker and the EF1α promoter was liberated from pDML190 by digestion with the restriction enzymes XbaI and ClaI.

Plasmid pDML200 was built by inserting three PCR products into pDML190, which was cut with BamHI and SpeI. The first PCR product corresponds to a short linker (GSG) followed by mNeonGreen (#2–177), which was PCR amplified from pDML190 with primers DML_P626_F and DML_P556_R. The second PCR product corresponds to the *K*.*l*. *URA3* marker, which was amplified form pDML190 with primers DML_P557_F and DML_P642_R. The third PCR product corresponds to mNeonGreen (#60–236) followed by another short linker (GSG), which were amplified from pDML190 with primers DML_P643_F and DML_P630_R.

Plasmid pDML219 was built by PCR amplifying a fragment that consists of mNeonGreen (#60–177), *K*.*l*. *URA3*, mNeonGreen (#60–236), and a stop codon (TAA) from pDML200 with primers DML_P712_F and DML_P713_R. The PCR product was then cloned into pDML152, which was digested with the restriction enzymes NcoI and SpeI.

Plasmid pDML222 was built by inserting two PCR products into pDML190, which was digested with ClaI and BlpI. The first PCR product corresponds to non-yeast codon-optimized mNeonGreen (amino acid #1–59), which was amplified from pDML63 with primers DML_P738_F and DML_P739_R. The second PCR product corresponds to yeast codon-optimized mNeonGreen (#60–236), which was amplified from pDML190 with primers DML_P740_F and DML_P609_R.

Plasmid pDML223 was built by inserting two PCR products into pDML219, which was cut with PacI and XbaI. The first PCR product corresponds to yeast codon-optimized mNeonGreen (#5–177), which was amplified form pDML219 with primers DML_P110_F and DML_P741_R. The second PCR product corresponds to non-yeast codon-optimized mNeonGreen (#178–236), which was amplified from pDML63 with primers DML_P742_F and DML_P743_R.

The plasmid pDH149 consists of a pFA6a vector backbone and four inserts: a linker (AGSASGGGG), the first 471 bp of mGFPmut3 after the first ATG (amino acid #2–158) that correspond to the N-terminal fragment of GFP (i.e. "NmGFPmut3", following the previously used notation [38]), the *ura4* cassette, and full-length mGFPmut3. Downstream of NmGFPmut3 are the *adh1* terminator, which is followed by the *ura4* cassette that contains the promoter, ORF, and terminator of *ura4*. The cassette containing the mGFPmut3 fragments with *ura4* can be isolated by restriction digestion of pDH149 with AfeI and BclI, if the plasmid is grown in a *dam- E*. *coli* strain.

Plasmid pDH165 has a similar layout to pDH149, but the *ura4* marker is substituted by concatenated kanMX6 [37] and HSV1tk [39] markers. The kanMX6 cassette contains the TEF1-alpha promoter and terminator, while HSV1tk cassette contains the *adh1* promoter and *nmt1* terminator.

Plasmid pDH193 is identical to pDH149 except that the BclI restriction site was replaced by a BamHI site, which is not blocked by *dam* methylation.

Plasmids pDH149, pDH165, pDH193, pDML20, pDML61, pDML99, pDML152, pDML190, pDML193, pDML200, pDML219, pDML222, and pDML223 were deposited with the Addgene Plasmid Repository.

### Yeast Strain Constructions

#### PCR amplification of the ≥300-bp long homology arms H1 and H2

The H1 and H2 homology arms were PCR amplified from yeast genomic DNA using the Q5 high-fidelity 2x master mix (NEB) and primers F1 and R1 and primers F2 and R2, respectively. The PCR condition was 98°C for 30 s, followed by 30 cycles of 98°C for 10 s, 53°C for 30 s, and 72°C for 20 s, and a final extension at 72°C for 2 min. The PCR products were then column-purified with the QIAquick PCR purification kit (Qiagen) and eluted in EB buffer. The primer sequences of the F1, R1, F2, and R2 primers for C-terminal tagging with a selection marker, scarless C-terminal tagging, and scarless N-terminal tagging of all *S*. *cerevisiae* ORFs are listed in S4 Table. The primer sequences for scarless C-terminal tagging of all *S*. *pombe* ORFs are listed in S5 Table.

#### Construction of C-terminal gene fusions with a selection marker in *S*. *cerevisiae*

*PRE1* was C-terminally tagged with mNeonGreen and the *C*.*g*. *TRP1* selection marker using plasmid pDML99 as the PCR template and a primer mix that contains the gene-specific H1 and H2 homology arms and the primers PRE1_CM_F1 and PRE1_CM_R2 (S4 Table). The primer mix was made by adding 25 μl 100 mM PRE1_CM_F1 primer, 25 μl 100 mM PRE1_CM_R2 primer, 250 ng of the H1 homology arm (i.e. PRE1_CM_H1), 250 ng of the H2 homology arm (i.e. PRE1_CM_H2), and *x* μl ddH_2_0 to a final volume of 100 μl. For the PCR reaction, 20 μl 2x Q5 master mix (NEB), 20 μl ddH_2_O, 2 μl PRE1_CM primer mix, and 100–200 ng of plasmid pDML99 were used. The PCR condition was 98°C for 30 s, followed by 30 cycles of 98°C for 10 s, 53°C for 30 s, and 72°C for 1.5 min, and a final extension at 72°C for 2 min. The integration cassette was transformed into *S*. *cerevisiae* strain 4436 and cells were plated on SD-trp plates. Correct chromosomal integration was confirmed by colony PCR using the gene-specific primer DML_P379_F and primer DML_P37_R, which binds to mNeonGreen.

Similarly, *TDH3* was C-terminally tagged with mNeonGreen and the *C*.*g*. *TRP1* marker using primers TDH3_CM_F1, TDH3_CM_R1, TDH3_CM_F2, and TDH3_CM_R2 (S4 Table).

#### Construction of scarless N-terminal gene fusions in *S*. *cerevisiae*

Scarless N-terminal tagging of *RSP5* with mNeonGreen or mCherry was done using pDML190 or pDML193, respectively, as the PCR template. Before the *K*.*l*. *URA3* marker was excised (using 5-FOA), *RSP5* was N-terminally tagged with partial mNeonGreen (amino acid #60–238) or mCherry (amino acid #60–236), which are non-fluorescent. The primer mix was made as described above except that primers RSP5_N_F1 and RSP5_N_R2, and homology arms RSP5_N_H1 and RSP5_N_H2 were used. The PCR was performed as described above. Several μg PCR product were transformed into yeast strain 4436 and the cells were spread on SD-ura plates. Colonies were re-struck on SD-ura plates. Correct chromosomal integration was confirmed by colony PCR with primers DML_P587_F and DML_P476_R (upstream flank of integration site) and primers DML_P584_F and DML_P588_R (downstream flank of integration site). To pop out the *URA3* marker and to reconstitute the mNeonGreen, an overnight culture of a PCR-verified clone was grown in YPD and 100 μl cell suspension was then spread on a 5-FOA plate. Resulting colonies were re-struck on a YPD plate and confirmed by colony PCR using primers DML_P74_F and DML_P75_R (mNeonGreen-*RSP5*). For strain yR-a-298, successful N-terminal tagging of *RSP5* with mNeonGreen was further confirmed by sequencing of the colony PCR band and Western blotting.

N-terminal tagging of *YPT1* with mNeonGreen or mCherry was done as described above but with primers YPT1_N_F1, YPT1_N_R1, YPT1_N_F2, and YPT1_N_R2. The yeast colonies were checked by PCR using primers DML_P368_F and DML_P476_R (upstream flank) or primers DML_P584_F and DML_P369_R (downstream flank), and with primers DML_P368_F and DM_P369_R after excision of the *URA3* marker (mNeonGreen-*YPT1*). N-terminal tagging of the other 10 Rab proteins was done analogously. We have not encountered a problem when we N-terminally tagged a protein with a partial fluorescent protein (e.g. mNeonGreen_60-236_). Though alternatively, plasmid pDML222 can be used as the PCR template, which would result in an N-terminally tagged protein with full-length mNeonGreen even prior to excision of the *K*.*l*. *URA3* marker (S1 Fig).

#### Construction of scarless C-terminal gene fusions in *S*. *cerevisiae*

Scarless C-terminal tagging was performed following the protocol described above for scarless N-terminal tagging except that plasmids pDML219 or pDML223 were used as the PCR template. For *PRE1*-mNeonGreen, plasmid pDML223 and primers PRE1_C_F1, PRE1_C_R1, PRE1_C_F2, and PRE1_C_R2 were used. The integration cassette was transformed into yeast strain 4436 followed by selection on SD-ura plates. Correct chromosomal integration of *PRE1*-linker-mNG_5-236_-*URA3*-mNG_60-236_ was confirmed with primers DML_P379_F and DML_P528_R (upstream flank) and primers DML_P504_R and DML_P380_R (downstream flank). Excision of the *URA3* marker and presence of *PRE1*-mNG was confirmed with primers DML_P379_F and DML_P380_R.

For *TDH3*-mNeonGreen, plasmid pDML219 and primers TDH3_C_F1, TDH3_C_R1, TDH3_C_F2, and TDH3_C_R2 were used. Correct chromosomal integration was confirmed with primers TDH3_C_Fc and DML_P744_R.

#### Construction of scarless C-terminal gene fusions in *S*. *pombe*

For scarless C-terminal tagging of the *tdh1* gene in *S*. *pombe*, the "linker-NmGFPmut3-*ura4*-mGFPmut3" cassette was prepared by overnight restriction digest of plasmid pDH149 with AfeI and BclI, followed by column purification. BclI is sensitive to *dam* methylation, hence *E*. *coli* strain ER2925 (NEB) was used to prepare pDH149. Two 900 bp-long homology arms for *tdh1* were PCR amplified from *S*. *pombe* genomic DNA using primers tdh1_F1 and thd1_R1 and primers tdh1_F2_and thd1_R2. The resulting DNA fragments contain 27 and 29 bp overlap with the linker and the 3' end of mGFPmut3, respectively. Using isothermal assembly [35], the homology arms were attached to the cassette from the restriction digestion (1:1:1 molar ratio) by incubation at 50°C for 45 minutes. A total of ~1.2 μg of the digestion product was used, and the reaction volume was 40 μl reaction (S2 Fig). The entire construct can be generated by PCR as described above, but due to higher reproducibility at the time, isothermal assembly was used. To use the cassette with the kanMX6 and HSV1tk markers in place of *ura4*, pDH165 was digested instead of pDH149 following an identical protocol.

For transformation of *S*. *pombe*, the standard lithium acetate method [34] was used. Briefly, *S*. *pombe* cells were grown in YES medium to OD_600_ ~0.5, harvested, resuspended in 100 μl of 0.1 M lithium acetate pH 4.9, and incubated at 30°C for 60 min. The whole amount of the isothermal assembly reaction (40 μl; without purification) was added to the cell suspension, then 290 μl of 50% (w/v) PEG4000 was added, and incubated at 30°C for 60 min. The cells were heat-shocked for 15 min at 43°C, centrifuged at 3,000 g, and the pellet was resuspended in sterile water followed by plating on either a PMG-ura or a YES+G418 (60 mg/L) plate. Surviving colonies were picked after 3 days and correct integration was confirmed by colony PCR using primers that encompassed inner and outer regions of the integrated cassette. The confirmed colonies were grown in 5 ml YES medium for 3 days to allow for homologous recombination between the NmGFPmut3 and the full-length mGFPmut3. From this culture, 60 μl was plated onto PMG+5FOA (0.1% w/v) or YES+FUdR (20 mg/L) plate, depending on the initial marker being *ura4* or kanMX6 with HSV1tk. The presence of full-length mGFPmut3 and then absence of the selection markers were both confirmed by colony PCR.

### Automated Primer Design

The *S*. *cerevisiae* ORF names and DNA sequences for the primer computations were obtained from the GenBank files of the 16 chromosomes (NC_001133, NC_001134, NC_001135, NC_001136, NC_001137, NC_001138, NC_001139, NC_001140, NC_001141, NC_001142, NC_001143, NC_001144, NC_001145, NC_001146, NC_001147, and NC_001148; 25-FEB-2013 release). The total number of ORFs used for the automated primer design was 5886. For *S*. *pombe*, the ORF names and sequences were obtained from the GenBank files of the three chromosomes (CU329670, CU329671, and CU329672; 10-JAN-2012 release) and the total number of ORFs used was 5154. The mitochondrial ORFs were excluded for both yeasts. The automated primer design code was developed using MATLAB (MathWorks). The size of the upstream and downstream search window was increased from 500 to 700 bp when we calculated the primers for *S*. *pombe*. The computed primer sequences are listed in S4 and S5 Tables, respectively. The primer name is composed of the ORF name, the tagging method (C, CM, or N), and the type of primer (F1, R1, F2, R2, and Fc). The sequence of the 5’ appendix of the R1 and F2 primers is indicated using uppercase letters. The Fc primer binds to the genomic locus upstream of the F1 primer and is used in combination with a reverse primer that binds to the GFP for colony PCR. The calculation of the melting temperature (*T*_*m*_) of the primers is based on a nearest-neighbor model of DNA/RNA duplex stability [40] and the enthalpy and entropy of helix formation were calculated from a table of nearest-neighbor values [41]. The *T*_*m*_ values of the primers were calculated with the following formula: $T_{m}=\frac{\Delta H}{\frac{\left(\Delta S-10.8\right)}{1000}+\frac{R}{1000}∙\ln\left(\frac{\left[primer\right]}{4}\right)}-273.15+16.6∙{log}_{10}(\left[Na\right])+c$ where Δ*H* is the enthalpy (kcal/mol), Δ*S* is the entropy (cal/K/mol), *R* is the universal gas constant (1.987 cal/K/mol), and *c* is an empirically determined correction factor (*c* = 0.1518°C). The correction factor is a simple offset to match the result of the *T*_*m*_ calculation using the formula from above with the *T*_*m*_ value determined with the Lasergene PrimerSelect program (DNASTAR). For the calculations, we used a primer concentration of 1 μM and a sodium concentration of 50 mM. The DNA sequences corresponding to the 5’ appendices were not included in the *T*_*m*_ calculations. Primer sequences longer than 60 nucleotides (nts) were truncated to 60 nts. Hairpin formation was assessed using MATLAB’s ‘oligoprop’ function of the Bioinformatics Toolbox. The respective m-files are contained in S1 File and are also available on GitHub (https://github.com/DXL38/scarless_gene_tagging_in_yeast).

### Yeast Whole-Cell Extraction and Anti-Rsp5 Western Blot

The equivalent of two ODs of exponentially growing *S*. *cerevisiae* cells were pelleted (2,500 g, 2 min) at room temperature. After aspiration of the supernatant, the cells were resuspended in 1 ml dH_2_O using a thermomixer at 25°C. 150 μl of lysis solution (1.85 N NaOH, 7.5% β-mercaptoethanol) was added to the cell suspension, which was briefly vortexed and incubated on ice for 15 min. Next, 150 μl of 55% Trichloroacetic acid (TCA) solution was added, briefly vortexed, and incubated on ice for 10 min. The mixture was then centrifuged for 15 min at maximum speed with a tabletop centrifuge cooled to 4°C. The supernatant was aspirated and the centrifugation was repeated for 5 min to thoroughly remove the remaining TCA. The pellet was resuspended in 100 μl of High Urea buffer (200 mM Tris-HCl pH 6.8, 8 M Urea, 5% SDS, a pinch of bromophenol blue, and 50 mM TCEP was added to the buffer right before use). The solution was mixed with a thermomixer (1,500 rpm, 10 min) at 50°C. The extracts were then frozen in liquid nitrogen and stored at -80°C.

For western blotting, 10 μl of yeast extract was loaded on a 4–12% gradient NuPAGE™ Novex™ 4–12% Bis-Tris protein gel in MOPS buffer (Life Technologies) and the gel was run at 200 V for 50 min. The transfer of the proteins to the nitrocellulose membrane was carried out using the iBLOT system (Life Technologies). The membrane was probed with an anti-Rsp5 antibody (rabbit, 1:5,000 dilution) and an anti-PGK1 antibody (1:10,000, mouse monoclonal, ThermoFisher:22C5D8). The recordings were taken digitally using a Bio-Rad ChemiDoc station.

### Fluorescence Microscopy

Overnight cultures of the *S*. *cerevisiae* cells were >100× diluted, grown to exponential phase at 30°C, and then imaged at room temperature on agar pads made with 2% agarose (SeaKem LE Agarose, Lonza) dissolved in SD-CSM medium. All microscopy experiments were performed on an inverted microscope (Nikon TI-E) equipped with a Perfect Focus unit (Nikon), a high-speed emission filter wheel (Finger Lakes Instrumentation), a CFI Plan Apo Lambda 100× oil objective (Nikon), and an Andor Clara CCD camera. Fluorescence images were acquired using a Spectra-X LED system (Lumencor), a quad band dichroic (FF410/504/582/669-Di01, Semrock), and appropriate excitation and emission filters: GFP excitation (485/25, Lumencor), GFP emission (FF01-525/30, Semrock), RFP excitation (FF01-560/25-25, Semrock), and RFP emission (FF01-607/36, Semrock). The microscope was controlled using MATLAB and SDKs provided by Nikon and Andor.

## Results

In this report, we describe two methods for endogenous gene tagging with fluorescent proteins in yeast: scarless C-terminal tagging (Fig 1B-i) and scarless N-terminal tagging (Fig 1B-ii).

### Scarless C-Terminal Gene Tagging in *S*. *cerevisiae*

We used a two-step PCR synthesis method to generate an integration cassette with ≥300-bp long homology arms (Fig 1C). First, two ≥300-bp long fragments that are homologous to the regions upstream and downstream of the integration site were made by PCR using primers F1 and R1, and primers F2 and R2, respectively. The two resulting homology fragments were named H1 and H2. The primers R1 and F2 each contain a 5’ appendix that is incorporated into H1 and H2, respectively, and functions as a primer in the second round of PCRs. Next, the fragments H1 and H2 were mixed with primers F1 and R2 and used to amplify the complete integration cassette from a plasmid template, which attaches the ≥300-bp long homology arms, H1 and H2, to the ends of the integration cassette (Fig 1C).

For scarless gene tagging with a fluorescent protein such as GFP, the integration cassette contains a *URA3* marker that is flanked by two GFPs, which are either partial or full-length, and share approximately 350 bp sequence homology directly surrounding the *URA3* marker (Fig 1B and S1 Fig). The PCR-assembled integration cassette with the ≥300-bp long homology regions was then transformed into yeast cells followed by plating on SD-ura to select for chromosomal integration events. Correct integration can optionally be verified by colony PCR using primers that are specific to the upstream or downstream region of the chromosomal integration site and to the integration cassette. A correct clone was identified and grown in rich medium without selection, to allow spontaneous recombination between the two GFP fragments resulting in loss of the *URA3* marker. After overnight growth, the cell culture was then spread on a 5-FOA plate to select for cells that produce the full-length GFP fusion of the protein of interest (Fig 1C). Unlike C-terminal gene tagging with a marker, the scarless C-terminal tagging method does not disrupt the endogenous 3’ UTR, which can be important for mRNA localization [13–16,42] and regulation of transcription [17].

**Fig 2:**
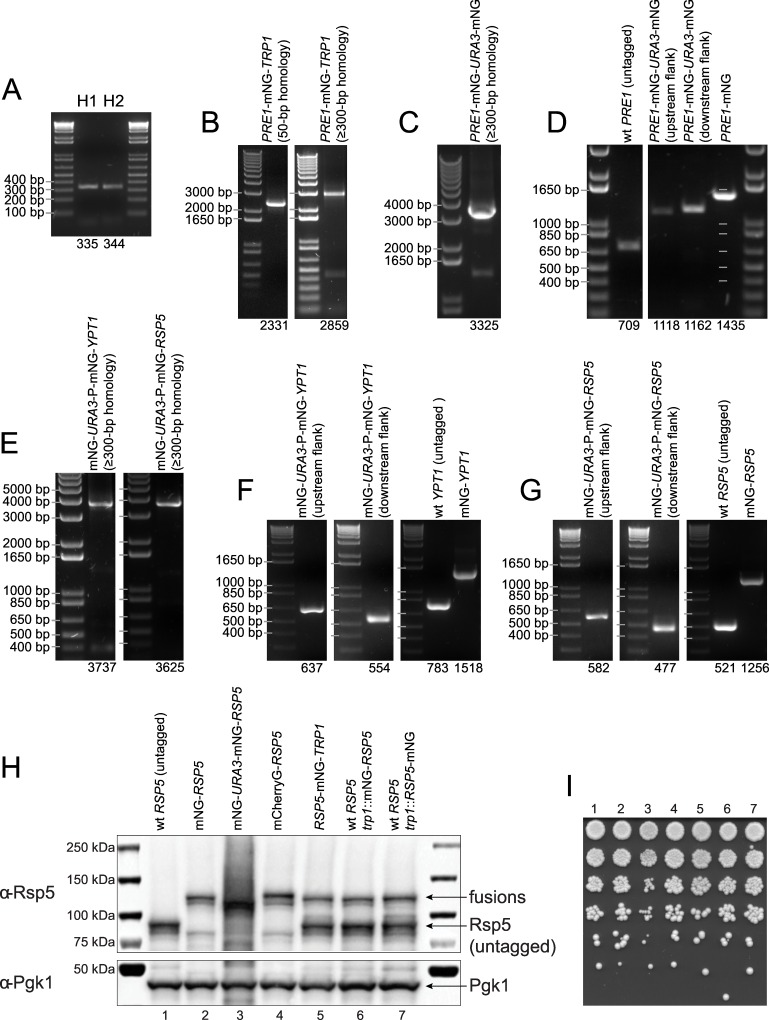
DNA gels and Western blot confirm strain construction. (A) PCR amplification of homology arms H1 and H2. (B) Integration cassettes for C-terminal tagging of *PRE1* with mNeonGreen (mNG) and the *TRP1* selection marker using either 50-bp or ≥300-bp homology. (C) Integration cassette for scarless C-terminal tagging of *PRE1* with mNeonGreen using ≥300-bp homology. (D) Colony PCR confirming correct chromosomal integration of the fusions at the *PRE1* gene locus. (E) Integration cassette for the scarless N-terminal tagging of *YPT1* and *RSP5* with mNeonGreen. (F) Colony PCR confirming correct integration of the mNG_1-177_-*URA3*-P_EF1α_-mNG_60-236_ construct at the *YPT1* gene locus with primer sets for the upstream (left) and downstream flank of the integration site (middle). Growth in rich medium followed by selection on 5-FOA leads to the identification of cells that have recombined the mNeonGreen fragments and lost the *URA3* marker, resulting in *YPT1* tagged N-terminally with mNeonGreen (right). Colony PCR of a strain with an unmodified, wild-type *YPT1* gene locus is shown as control. (G) Colony PCR confirms correct integration of the mNG_1-177_-*URA3*-P_EF1α_-mNG_60-236_ construct at the *RSP5* gene locus (left and middle). Recombination results in full-length mNeonGreen-*RSP5* at the endogenous gene locus (right). (H) Western blot analysis using an anti-Rsp5 antibody (upper) with *S*. *cerevisiae* strains expressing untagged Rsp5 (lane #1), mNeonGreen-Rsp5 (lane #2), mNeonGreen_60-236_-Rsp5 (lane #3, before *URA3* excision), mCherry-Rsp5 (lane #4), Rsp5-mNeonGreen (lane #5), untagged Rsp5 and an ectopic copy of mNeonGreen-Rsp5 (lane #6), and untagged Rsp5 and an ectopic copy of Rsp5-mNeonGreen (lane #7). The mNeonGreen_60-236_-Rsp5 fusion displays extensive self-ubiquitination, likely because of the partial mNeonGreen tag. The C-terminal Rsp5 shows a truncation product that most likely corresponds to un-tagged Rsp5. This truncation product is not observed with the N-terminal Rps5 fusions, which appear fully functional. The molecular weights are 92 kDa for untagged, wild-type Rsp5 and ~119 kDa for the fluorescent protein-tagged Rsp5 fusions. Pgk1 is used as loading control (lower). (I) Spotting assay with the strains that were used for the Western blot of Fig 2H.

As previously reported for *S*. *cerevisiae* [25], a higher transformation efficiency can be achieved by using an integration cassette with a longer region of homology to the integration site. We found that ≥300-bp homology increases the transformation efficiency substantially and streamlines strain construction considerably. For example, we C-terminally tagged the essential gene *PRE1*, which codes for the beta 4 subunit of the 20S proteasome, with mNeonGreen and the *TRP1* selection marker using an integration cassette with either 50-bp or ≥300-bp long homology arms (Fig 2A and 2B). Transformation with equal amounts of the integration cassettes (1 μg DNA) resulted in over 10× more correct clones with the ≥300-bp homology (50-bp homology: 3 clones in total, 2 out of 3 clones tested were correct; ≥300-bp homology: 42 clones in total, 24 out of 24 clones tested were correct). Thus, with the higher transformation yield and targeting efficiency, colony screening is essentially unnecessary when an integration cassette with ≥300-bp homology is used. We used W303 as the main strain background for this study but the tagging method has also been successfully carried out with other lab strain backgrounds (e.g. S288C) using the standard suite of auxotrophic or antibiotic selection markers. Pleiotropic yeast strains (e.g. wild yeast isolates) were successfully tagged with e.g. the G418 marker (data not shown).

**Fig 3:**
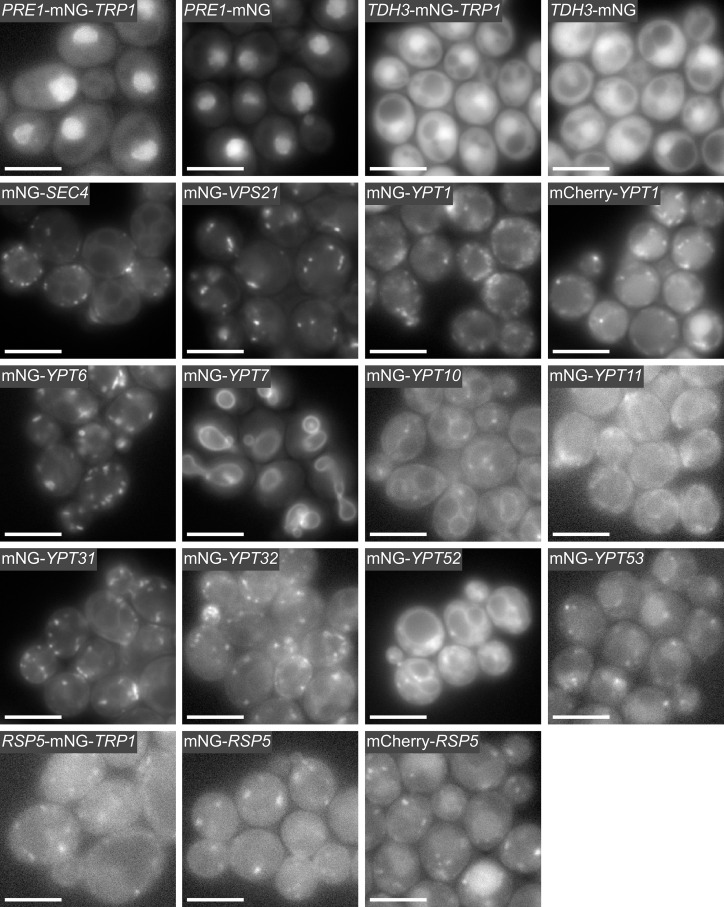
Fluorescence microscopy of *S*. *cerevisiae* cells producing mNeonGreen-tagged fusion proteins. Yeast strains were constructed by modifying the endogenous gene locus using C-terminal gene tagging with a marker (Fig 1A-ii), scarless C-terminal gene tagging (Fig 1B-i), or scarless N-terminal gene tagging (Fig 1B-ii). *PRE1* and *TDH3* were tagged C-terminally with mNeonGreen (mNG) using the *TRP1* selection marker or scarless. The eleven yeast Rab proteins (i.e. Sec4, Vps21, Ypt1, Ypt6, Ypt7, Ypt10, Ypt11, Ypt31, Ypt32, Ypt52, and Ypt53) are prenylated at their C-termini (S3 Fig) and were tagged N-terminally with mNeonGreen using the scarless gene tagging method. The Rab proteins localize to their expected organelles: for example Golgi (Ypt1, Ytp6), trans-Golgi network (Ypt6), vacuole and late endosomes (Ypt7), early endosomes (Vps21), recycling endosomes and post-Golgi exocytic vesicles (Ypt31), and secretory vesicles (Sec4). The ubiquitin ligase *RSP5* was tagged C-terminally and scarless N-terminally with mNeonGreen. The C-terminal *RSP5*-mNeonGreen fusion resulted in cell size enlargement (S4 Fig) but no growth phenotype was observed (Fig 2I and S5 Fig). The large-cell phenotype was not observed with the scarless N-terminal fusions. The cells producing mCherry-Rsp5 and mCherry-Ypt1 fusions look similar to the cells producing the corresponding N-terminal mNeonGreen fusions, although the mCherry fusions also display some vacuolar localization. *PRE1*, *YPT1*, *SEC4*, and *RSP5* are essential genes. Scale bar (white) is 5 μm.

To illustrate scarless C-terminal tagging in *S*. *cerevisiae*, we tagged *PRE1* with mNeonGreen (Fig 2C and 2D). Tagging *PRE1* with the integration cassette with the partial mNeonGreen tag was unsuccessful, most likely because the partial tag interfered with the function of the Pre1 protein. However, we obtained viable clones using a modified cassette with the full-length mNeonGreen tag (S1 Fig). Fluorescence microscopy of live *S*. *cerevisiae* yeast cells producing the Pre1-mNeonGreen fusion showed constitutive nuclear localization for Pre1 (Fig 3), which is the expected localization [43]. For the non-essential gene *TDH3*, we successfully performed scarless C-terminal tagging with either the partial or full-length mNeonGreen containing integration cassette as shown by imaging (Fig 3).

### Scarless N-Terminal Gene Tagging in *S*. *cerevisiae*

We successfully used the scarless N-terminal tagging method to tag the eleven *S*. *cerevisiae* Rab proteins (Sec4, Vps21, Ypt1, Ypt6, Ypt7, Ypt10, Ypt11, Ypt31, Ypt32, Ypt52, and Ypt53) and the ubiquitin ligase Rsp5 with mNeonGreen. As discussed above, the yeast Rab proteins are prenylated at two C-terminal cysteine residues (S3 Fig) and this prenylation is required for the attachment of the Rab proteins to lipid membranes [22]. We PCR generated the N-terminal integration cassettes and performed the positive and negative selection as described above (Fig 2E and 2F). Unlike the scarless C-terminal gene tagging method, the integration cassette for the scarless N-terminal tagging contains a constitutive promoter (P_EF1α_) just upstream of the second mNeonGreen fragment. After chromosomal integration and while the *URA3* marker is still in place, this promoter drives expression of a non-fluorescent N-terminal mNeonGreen fusion protein that is composed of only the second fragment of mNeonGreen and the protein being tagged. This additional feature was necessary for the tagging of essential genes like *SEC4*, *YPT1*, and *RSP5*. Recombination between the two mNeonGreen fragments excises the *URA3* marker and the EF1α promoter, and reconstitutes a full-length N-terminal mNeonGreen tag. Fluorescence microscopy of the Rab proteins revealed unique localization patterns consistent with proteins that are tethered to membranes and are involved in the secretory and endocytic pathways (Fig 3) [44]. Time-lapse imaging of the mNeonGreen-*YPT1* yeast strain showed that the mNeonGreen-Ypt1 protein is localized in ⪆20 diffraction-limited dots per cell, which are highly dynamic reflecting Ypt1’s role in vesicle trafficking (Fig 3 and S1 Movie).

It has previously been reported that the ubiquitin ligase Rsp5 (NEDD4 homolog) cannot be tagged C-terminally [24]. We were able to tag Rsp5 at its C-terminus with mNeonGreen, but the resulting strain showed a severe phenotype with an enlarged cell size (S4 Fig). We then constructed an N-terminal *RSP5* fusion with mNeonGreen using the scarless N-terminal tagging method (Fig 2E and 2G) and we found that the mNeonGreen-*RSP5* fusion did not show the aberrant cell morphology phenotype that we observed previously with the C-terminal fusion. Cell volume quantification further suggests that the N-terminal mNeonGreen-Rsp5 fusion is fully functional (S4 Fig). Time-lapse microscopy of *S*. *cerevisiae* cells expressing the mNeonGreen-*RSP5* fusion revealed cytoplasmic punctae, which exhibited some movement and often appeared adjacent to the vacuole (Fig 3 and S2 Movie). Western blotting of the various Rsp5 fusions with an anti-Rsp5 antibody confirmed that fusion proteins of the correct size were produced; some proteolytic cleavage was observed for the C-terminal fusion, but not for the N-terminal mNeonGreen-Rsp5 protein fusion (Fig 2H).

### Scarless C-Terminal Gene Tagging in *S*. *pombe*

**Fig 4:**
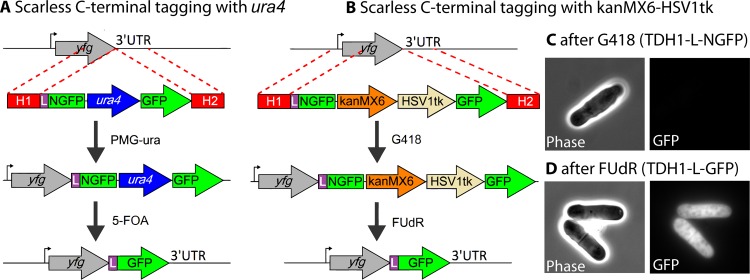
Scarless C-terminal tagging of a gene of interest in *S*. *pombe*. (A) Homology arms (H1 and H2) targeting a gene of interest were attached to a DNA cassette comprising a linker (L), NmGFPmut3 (NGFP), the *ura4* marker, and mGFPmut3 (GFP). The cassette was transformed into *S*. *pombe* cells followed by selection on a PMG-ura plate. The surviving colonies were cultured in YES medium for 3 days to allow spontaneous recombination between the GFP fragments and then plated on 5-FOA to select for cells that have *yfg* tagged scarless with linker-mGFPmut3. (B) The same method described in panel a, except that kanMX6 and HSV1tk were used as positive and negative selection markers, respectively. (C) After selection with G418, *tdh1* was tagged with NmGFPmut3, which is non-fluorescent and hence no fluorescence was detected by microscopy. (D) After 3 days of culture in YES medium, followed by selection with FUdR, cells with the recombined full-length mGFPmut3 protein attached to *yfg* showed the expected GFP signal. Scale bar (white) is 5 μm.

We also demonstrated the scarless C-terminal gene tagging method in *S*. *pombe* by tagging *tdh1* with mGFPmut3, which is a monomeric version of GFPmut3 as a result of the A206K mutation [45]. First, we constructed a plasmid that contains a linker sequence and “NmGFPmut3”, which is the mGFPmut3 equivalent of the N-terminal fragment of 'split-GFP' [38], followed by the *ura4* marker, and full-length mGFPmut3 (Fig 4A). Homology arms targeting the C-terminus of the *tdh1* gene (900 bp each) were PCR-amplified and attached to the linker-NmGFPmut3-*ura4*-mGFPmut3 fragment (see Materials and Methods, and S2 Fig). The assembled cassette was then transformed into *S*. *pombe* followed by selection on a PMG-ura plate. The resulting colonies were GFP negative, as expected, since NmGFPmut3 is non-fluorescent. After recombination between the mGFPmut3 fragments and loss of the *ura4* marker, GFP-positive colonies were observed (data not shown).

The *ura4* gene is convenient since it can serve as a positive and negative selection marker, which is a critical aspect of the scarless integration method. However, when *ura4* is used, the final strain with the mGFPmut3 tag is auxotrophic, which potentially can limit experiments that require a prototrophic strain. To circumvent this problem, we substituted the *ura4* marker with the kanMX6 and HSV1tk cassettes. These genes then serve as positive and negative selection markers, respectively, so that a prototrophic strain can be used. The kanMX6 gene product confers resistance to G418 while the viral thymidine kinase HSV1tk renders yeast sensitive to the halogenated thymidine derivative, FUdR (Fig 4B). We tested the *adh1* promoter and the SV40 promoter for driving the expression of the HSV1tk gene, and found that the former was strong enough, while the latter was too weak, to confer FUdR sensitivity. Similar to the case of the *ura4* cassette, the homology arms targeting the C-terminus of *tdh1* were attached to the linker-NmGFPmut3-kanMX6-HSV1tk-mGFPmut3 fragment. After selection with G418, the cells were grown in rich medium to allow recombination to occur. After a second selection on a FUdR-containing plate, the surviving *S*. *pombe* colonies, as well as individual cells, were all GFP positive (Fig 4C and 4D).

### Automated Primer Design for Long Homology Arms

The ≥300-bp homology method used here requires four primers (i.e. F1, R1, F2, and R2) for tagging a gene of interest. The location of the R1 and F2 primer-binding site determines the chromosomal integration site, while the length of the two homology arms is determined by the binding site of primers F1 and R2, respectively, and can hence be increased if longer homology is needed (Fig 1C). Since the four primers are ≤60 nucleotides in length, the two-step PCR synthesis is more cost-effective than direct gene synthesis of long oligonucleotides (e.g. using gBlocks from Integrated DNA Technologies).

**Fig 5:**
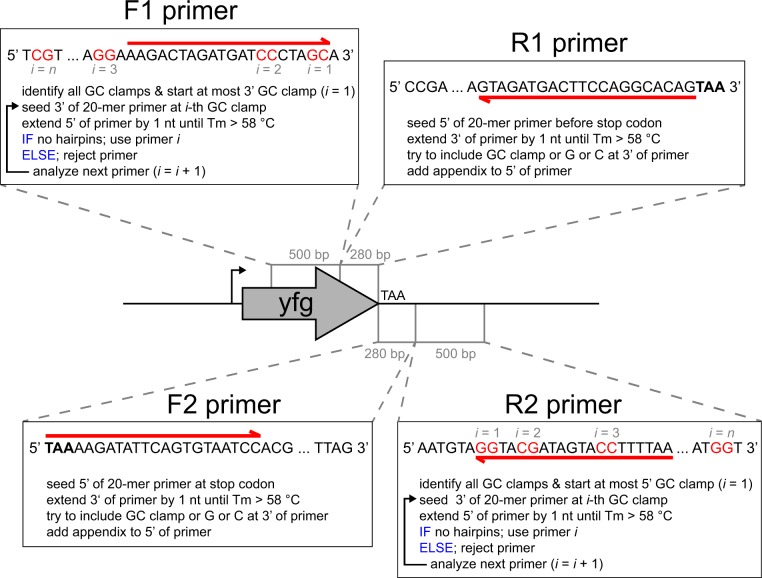
Pseudocode describing the automated primer design algorithm. The illustration depicts the primer design for C-terminal gene tagging of a gene of interest (yfg) with a fluorescent protein. N-terminal and internal gene tagging is analogous except for the insertion site. The primers are shown as arrows (red) above and below the respective DNA sequences. GC clamps in the 500-bp upstream and downstream search regions are highlighted in red. The primer sequences for scarless N-terminal, scarless C-terminal, and C-terminal gene tagging with a marker for essentially all *S*. *cerevisiae* ORFs are provided in S4 Table. The primer sequences for scarless C-terminal tagging of all *S*. *pombe* ORFs are provided in S5 Table. The code for the automated primer design is contained in S1 File and available on GitHub (https://github.com/DXL38/scarless_gene_tagging_in_yeast). Primers F1 and R1 are used for amplifying the homology arm H1, and primers F2 and R2 are used for amplifying the homology arm H2. The homology arms H1 and H2 are ≥300 bp in size.

To further increase the throughput of strain construction, we developed an automated primer design algorithm (Fig 5) and employed the code to calculate primers for all 5886 ORFs of *S*. *cerevisiae*. For the C-terminal tagging methods, the primer sequences for 5866 ORFs are provided (S4 Table). Primers could not be designed for the following twenty ORFs because their respective C-termini are located too close to the telomeres: YBL113C, YCR108C, *YRF1-1*, YIL177C, YEL077C, *YRF1-2*, YFL068W, YFR057W, *YRF1-3*, YHL050C, YHR219W, YJL225C, YLL067C, *YRF1-5*, YML133C, *YRF1-6*, YOL166W-A, *YRF1-8*, *YRF1-7*, and YPR204W (the table entry is “N/A” for those ORFs). We also computed the primer sequences for scarless N-terminal tagging of 5882 *S*. *cerevisiae* ORFs (S4 Table). Primers could not be designed for the following four ORFs because their respective N-termini are located too close to the telomeres: YCR108C, YER190C-B, YFL068W, and YOL166W-A. We also used the code to compute primer sequences for scarless C-terminal tagging of the 5154 ORFs of *S*. *pombe* (S5 Table). We were not able to design primers for *tlh1*, SPAC750.08c, and SPBC1348.15 because of their proximity to the telomeres. We further adapted the code to *E*. *coli* for C-terminal gene tagging using a selection marker (data not show). The primer design algorithm can also be easily adapted to other microbes for which genome sequences are available.

## Discussion

In this paper, we describe a method for the construction of scarless gene fusions with fluorescent proteins in budding and fission yeast using a one-step transformation followed by a two-step selection. Our method is reliable, low-cost, and highly efficient, avoiding the time-consuming second transformation step, which was required in previous methods for scarless integration [46,47]. Further, the method described here uses long homology arms, which results in a much higher transformation efficiency and requires essential no (or very little) screening to identify a correct clone. Finally, the time-consuming primer design has been fully automated and the primer sequences for all *S*. *cerevisiae* and *S*. *pombe* ORFs are provided (S4 and S5 Tables).

We also attempted scarless internal tagging (Fig 1B-iii), but we were not able to obtain working fusions, which we believe was because of the choice of the integration site. We attempted to build an internal *RSP5* fusion by inserting mNeonGreen either after Rsp5 amino acid #142 or #442 in a diploid yeast strain, but were not able to obtain working fusions, suggesting that the choice of the insertion sites requires further optimization. The first insertion site was between the C2 and WW1 domains and the second insertion site was between the WW3 and the HECT domains. We envision that a viable internal integration site for *RSP5* could be found using a recently developed method that employs an *in vitro* Tn7-transposon mutagenesis system for random insertions of the tag into the protein of interest [48], followed by screening for cell viability using a simple growth assay.

Many yeast proteins are difficult to detect by fluorescence microscopy because of low expression levels and high cellular autofluorescence. To overcome these limitations, we used mNeonGreen [30] for the gene tagging in *S*. *cerevisiae* since mNeonGreen is currently the brightest fluorescent protein available. Additionally, we found, for *S*. *cerevisiae*, that codon optimization resulted in an mNeonGreen variant that is significantly brighter than non-yeast codon-optimized mNeonGreen (S6 Fig). We believe that many yeast imaging studies will benefit greatly from using yeast codon-optimized mNeonGreen as the first choice fluorescent protein especially for visualizing low-abundance proteins. The yeast codon-optimized mNeonGreen tagging plasmids for *S*. *cerevisiae* were deposited with the Addgene plasmid repository.

The automated primer design algorithm that we developed facilitated small-scale and high-throughput gene tagging in the yeasts *S*. *cerevisiae* and *S*. *pombe*. The code can be easily adapted to other organisms, like cyanobacteria, which require integration cassettes with longer flanking regions for the construction of endogenous gene fusions by homologous recombination [37,49]. To adapt the entire scarless gene tagging method to another microbe, the availability of good positive and negative selectable markers is indispensable and needs to be ensured.

The scarless gene tagging methods described in this report are easy to perform and produce very robust results. We envision that these methods will be widely used for the construction of scarless gene fusions in yeast and other organisms.

## Supporting Information

S1 Fig Partial GFP *vs*. full-length GFP tag for scarless gene tagging in *S*. *cerevisiae*. Cartoons depicting scarless C-terminal and N-terminal tagging of a gene of interest (yfg) using an integration cassette with either a partial GFP tag or full-length GFP (left and right, respectively). Use of an integration cassette with the partial GFP tag results in a non-fluorescent intermediate fusion protein (prior to excision of the *URA3* marker), which becomes fluorescent after recombination of the two GFP parts. (A) Scarless C-terminal gene tagging of the essential *PRE1* gene was only successful using the full-length mNeonGreen-*URA3*-mNeonGreen_60-238_ integration cassette (right), possibly because the partial mNeonGreen tag (amino acid #5–177) interfered with the function of the protein. *PRE1* can be tagged in one step with full-length mNeonGreen using a selection marker (Figs 2 and 3). (B) Scarless N-terminal gene tagging with partial or full-length GFP tags. PCR amplification of the integration cassettes from the plasmid templates requires unique primer-binding sites. For scarless C-terminal tagging with full-length GFP, the forward primer (part of H1) binds to the linker (purple) of the first GFP and the reverse primer (part of H2) binds to the C-terminus of the second GFP. The reverse primer-binding site is unique because the C-terminus of the first GFP (dark green) uses different codons (i.e. non-yeast codon-optimized). For scarless N-terminal tagging with full-length GFP, the forward primer (part of H1) binds to the N-terminus of the first GFP, which is unique because the N-terminus of the second GFP (dark green) uses different codons (i.e. non-yeast codon-optimized). The reverse primer (part of H2) binds to the linker (purple) sequence. Unique primer binding sites are crucial for efficient PCR amplification of the integration cassette.
(EPS)

S2 Fig Isothermal assembly of the integration cassette for scarless C-terminal gene tagging in *S*. *pombe*. (A) Plasmid pDH149 was digested with AfeI and BclI to liberate the 3228 bp insert, which corresponds to linker (in purple)-NmGFPmut3-ura4-mGFPmut3. The insert and the 900-bp-long homology arms H1 and H2 (red), which have 27 and 29 bp overlap (purple and dark green) with the insert, respectively, were attached using isothermal assembly. H1 and H2 have homology with the C-terminal part of yfg and the 3’ UTR of yfg, respectively. (B) The isothermal assembly reaction was run for varying times from 10 min to ~90 min, and loaded onto a DNA gel. After a 45-min reaction, the predominant product was 5 kb long, which corresponds to the assembled integration cassette containing both homology arms and the insert, while the bands corresponding to the single homology arm with the insert (4 kb) or the insert-only (3 kb) are negligible.
(EPS)

S3 Fig Depiction of the amino acid sequences of the eleven Rab proteins of *S*. *cerevisiae*. The first five N-terminal and the last ten C-terminal amino acids of the Rab GTPases are shown. The two C-terminal cysteine residues (blue) are the prenylation sites, which are required for lipid attachment of the Rab proteins.
(EPS)

S4 FigEffect of expression of N- and C-terminal *RSP5* fusions with mNeonGreen on the volume of *S*. *cerevisiae* cells.Single-cell imaging and segmentation was performed as described below (see caption of S6 Fig). The major and minor axes of each yeast cell were measured from the segmentation mask and used for calculating the cell volume, which was approximated as a prolate spheroid using the following formula: $volume=\frac{4}{3}∙\pi ∙\frac{major\;axis}{2}{∙(\frac{minor\;axis}{2})}^{2}$. Wild-type haploid and diploid yeast cells have an average cell volume of approximately 45 and 90 μm^3^, respectively. When the essential *RSP5* gene is C-terminally tagged with mNeonGreen (mNG) at the endogenous gene locus in the haploid background, the yeast cells show a large cell phenotype with a cell volume similar to the diploid. The C-terminal *RSP5*-mNeonGreen fusion appears to have a weak dominant negative effect since haploid cells that express *RSP5*-mNeonGreen as a second copy (integrated in the *trp1* locus) and have the endogenous *RSP5* untagged, have a somewhat smaller cell volume. The N-terminal mNeonGreen-*RSP5* fusion does not increase the cell volume, which strongly suggests that this fusion is functional.(EPS)

S5 Fig Growth curves of *S*. *cerevisiae* strains expressing *RSP5* fusions or the N-terminal *YPT1* fusion with mNeonGreen reveal no growth phenotype. Yeast cells were grown in YPD medium in 384-well plates at 25°C without agitation. The OD_600_ was measured using a Multiskan Go plate reader (ThermoFisher Scientific). (A) Each strain was grown in 8 different wells and individual growth curves were plotted for visualization. Logarithmic fits were performed using a lower bound of OD_600_ = 0.01 and an upper bound of OD_600_ = 0.6. The part of the growth curve that was used for the fit is highlighted in light green. The first row corresponds to strain #1 (4436), second row to strain #2 (yR-a-2), third row to strain #3 (yR-a-60), fourth row to strain #4 (yR-a-298), fifth row to strain #5 (yR-a-299), and sixth row to strain #6 (yDML226). (B) The bar plot shows the averaged doubling time of the eight replicates for the six different strains (i.e. #1 –#6). The error bars represent one standard deviation from the mean. All strains show relatively similar doubling times and no strain displays a significant growth defect.
(EPS)

S6 Fig Yeast codon optimization of mNeonGreen leads to enhancement of fluorescence signal for microscopy. The fluorescence intensities of protein fusions constructed in *S*. *cerevisiae* with either non-yeast codon-optimized mNeonGreen or yeast codon-optimized mNeonGreen were compared. Yeast codon optimization of mNeonGreen increases the brightness of the corresponding fusion proteins in *S*. *cerevisiae*. The *TDH1* and *PRE1* genes were C-terminally tagged at their endogenous loci with either normal mNeonGreen or yeast codon-optimized mNeonGreen. The resulting four haploid yeast strains were grown in minimal medium (SD-CSM) and the cells were imaged on agar pads. The yeast cells also expressed a cytoplasmic mCherry (RFP) marker that was used for segmentation of the microscopy image. For image analysis, the resulting cell mask was overlaid on the GFP image and the average fluorescence intensity (i.e. average pixel value intensity) was calculated for each individual cell. Between 700 and 3,000 yeast cells were analyzed for each strain and the data is displayed as histograms. The number of cells (N), brightest cell (max), dimmest cell (min), mean GFP fluorescence of the cells (mean), median GFP fluorescence of the cells (median), GFP fluorescence of the most frequent cell (mode), and the standard deviation (σ) and coefficient of variation (CV) of the cell population are displayed. Codon optimization increases the brightness of the mNeonGreen fusion proteins by > 50%. A similar effect was also observed for other yeast codon-optimized fluorescent proteins such as mCherry or msfGFP (data not shown).
(EPS)

S1 File MATLAB code (m-files) for automated primer design. (ZIP)

S1 Movie Time-lapse movie of *S*. *cerevisiae* cells expressing the mNeonGreen-*YPT1* fusion. The scale bar (white) corresponds to 5 μm.
(MOV)

S2 Movie Time-lapse movie of *S*. *cerevisiae* cells expressing the mNeonGreen-*RSP5* fusion. The scale bar (white) corresponds to 5 μm.
(MOV)

S1 Table *S*. *cerevisiae* and *S*. *pombe* strains used in this study. (PDF)

S2 Table *E*. *coli* plasmids used in this study. (PDF)

S3 Table Primers used in this study. (PDF)

S4 Table Primer sequences for *S*. *cerevisiae* gene tagging. Primer sequences for tagging every *S*. *cerevisiae* ORF at the N-terminus using the scarless tagging method (name tag: N), at the C-terminus using the scarless tagging method (name tag: C), and at the C-terminus using a selection marker (name tag: CM). The primer name is composed of three parts separated by underscores: the gene name, the tagging method (i.e. N, C, or CM), and the type of primer (i.e. F1, R1, F2, R2, or Fc). The Fc primer is used to confirm correct chromosomal integration by colony PCR.
(XLSX)

S5 Table Primer sequences for *S*. *pombe* gene tagging. Primer sequences for tagging every *S*. *pombe* ORF at the C-terminus using the scarless tagging method. Naming system used is identical to S4 Table.
(XLSX)
