# Supplementary material for: Scarless Gene Tagging with One-Step Transformation and Two-Step Selection in Saccharomyces cerevisiae and Schizosaccharomyces pombe
Source: PLoS One. 2016 Oct 13;11(10):e0163950. doi: 10.1371/journal.pone.0163950 (PMC5063382; doi:10.1371/journal.pone.0163950)
Supplement: S1 Table — (PDF) [file pone.0163950.s010.pdf]

S1 Table. ***S. cerevisiae*** **and** ***S. pombe*** **strains used in this study.**

Strain	Description	Marker	Reference
4436	W303 *MATa can1-100 his3-11,15 leu2-3,112 ura3-1 trp1-1*	-	Lab collection
4437	W303 *MATα can1-100 his3-11,15 leu2-3,112 ura3-1 trp1-1*	-	Lab collection
4437-LEU2	W303 *MATα can1-100 his3-11,15 leu2::*pNH605 *ura3-1 trp1-1*	*LEU2*	This study
yDML77	4436 *TRP1 PRE1*-mNeonGreen (non-yeast codon-optimized)-T_{ADH1}-*C.g. HIS3*	*TRP1*, *HIS3*	This study
yDML78	4436 *TRP1 PRE1*-mNeonGreen-T_{ADH1}-*C.g. HIS3*	*TRP1*, *HIS3*	This study
yDML226	4437 mCherry-*RSP5*	-	This study
yDML231	4437 mCherry-*YPT1*	-	This study
yDML242	W303 *MATa/MATα can1-100/can1-100 his3-11,15/his3-11,15 leu2-3,112/leu2::*pNH605 *ura3-1/ ura3-1 trp1::*pNH604/ *trp1-1*	*LEU2*, *TRP1*	This study
yDML248	4436 P_{SEC4}-mNeonGreen-*SEC4*	-	This study
yDML249	4436 P_{YPT6}-mNeonGreen-*YPT6*	-	This study
yDML250	4436 P_{YPT7}-mNeonGreen-*YPT7*	-	This study
yDML251	4436 P_{YPT10}-mNeonGreen-*YPT10*	-	This study
yDML252	4436 P_{YPT11}-mNeonGreen-*YPT11*	-	This study
yDML253	4436 P_{YPT31}-mNeonGreen-*YPT31*	-	This study
yDML254	4436 P_{YPT32}-mNeonGreen-*YPT32*	-	This study
yDML255	4436 P_{YPT52}-mNeonGreen-*YPT52*	-	This study
yDML256	4436 P_{YPT53}-mNeonGreen-*YPT53*	-	This study
yDML260	yR-a-60 *HO*::pDML112	NAT	This study
yDML261	4436 *trp1::*pNH604-P_{RSP5}-*RSP5*-mNeonGreen *HO::*pDML112	*TRP1*, NAT	This study
yDML262	4436 *trp1::*pNH604-P_{RSP5}-mNeonGreen-*RSP5* *HO::*pDML112	*TRP1*, NAT	This study
yDML263	yR-a-298 *HO::*pDML112		This study
yDML264	4436 P_{VPS21}-mNeonGreen-*VPS21*	-	This study
yDML265	4436 *TDH3*-mNeonGreen	-	This study
yDML267	4436 *PRE1*-mNeonGreen	-	This study
yR-a-2	4436 *trp1::*pNH604	*TRP1*	This study
yR-a-47	4436 *PRE1*-mNeonGreen-T_{ADH1}-*C.g. TRP1*	*TRP1*	This study
yR-a-60	4436 *RSP5*-mNeonGreen-T_{ADH1}-*C.g. TRP1*	*TRP1*	This study
yR-a-136	4436 *TDH3*-mNeonGreen-T_{ADH1}-*C.g. TRP1*	*TRP1*	This study
yR-a-298	4436 *trp1::*pNH604 P_{RSP5}-mNeonGreen-*RSP5*	*TRP1*	This study
yR-a-299	4436 *trp1*::pNH604 P_{YPT1}-mNeonGreen-*YPT1*	*TRP1*	This study

FWP10	*ura4-294, h-*	-	Winston lab
FWP172	*ade6-m210, leu1-32, ura4-D18, h-*	-	Winston lab
DH0	*ade6::ade6+, leu1-32, ura4-D18, h-*	*ade6*	This study
DH159	*tdh1*-NmGFPmut3-*ura4*-mGFPmut3,*ura4-D18, leu1-32, h-*	*ura4*	This study
DH160	*tdh1*-mGFPmut3, *ura4-D18, leu1-32, h-*	*ura4*	This study
DH178	*tdh1*- NmGFPmut3-kanMX6-HSV1tk-mGFPmut3, *ura4-294, h-*	kanMX6	This study
DH179	*tdh1*-mGFPmut3, *ura4-294, h-*	-	This study
