# Supplementary material for: Scarless Gene Tagging with One-Step Transformation and Two-Step Selection in Saccharomyces cerevisiae and Schizosaccharomyces pombe
Source: PLoS One. 2016 Oct 13;11(10):e0163950. doi: 10.1371/journal.pone.0163950 (PMC5063382; doi:10.1371/journal.pone.0163950)
Supplement: S2 Table — (PDF) [file pone.0163950.s011.pdf]

S2 Table. *E. coli* plasmids used in this study.

Plasmid	Description	Marker	Reference
pDML20	pFA6a-linker(5xGA)-msfGFP-T_{ADH1}-kanMX6	Amp; G418	This study
pDML61	pFA6a-linker(5xGA)-msfGFP-T_{ADH1}-*C.g. HIS3*	Amp; *HIS3*	This study
pDML63	pFA6a-linker(5xGA)-mNeonGreen (non-yeast codon-optimized)-T_{ADH1}-*C.g. HIS3*	Amp; *HIS3*	This study
pDML99	pFA6a-linker(5xGA)-mNeonGreen-T_{ADH1}-*C.g. HIS3*	Amp; *HIS3*	This study
pDML112	pFA6-*HO*-P_{TDH3}-mCherry -T_{ADH1}-NAT-*HO*	Amp; NAT	This study
pDML145	pNH604-P_{TDH3}-mNeonGreen-T_{ADH1}	Amp; *TRP1*	This study
pDML152	pFA6a-linker(5xGA)-mNeonGreen-T_{ADH1}-*C.g. TRP1*	Amp; *TRP1*	This study
pDML166	pNH604-$P_{EF1\alpha}$-mNeonGreen-T_{ADH1}	Amp; *TRP1*	This study
pDML190	pFA6a-mNeonGreen$_{(1\text{-}177)}$-*K.l. URA3*-$P_{EF1\alpha}$-M-mNeonGreen$_{(60\text{-}236)}$-linker(5xGA)	Amp; *URA3*	This study
pDML193	pFA6a-mCherry$_{(1\text{-}177)}$-*K.l. URA3*-$P_{EF1\alpha}$-M-mCherry$_{(60\text{-}236)}$-linker(5xGA)	Amp; *URA3*	This study
pDML200	pFA6a-GSG-mNeonGreen$_{(3\text{-}177)}$-*K.l. URA3*-mNeonGreen$_{(60\text{-}236)}$-GSG	Amp; *URA3*	This study
pDML219	pFA6a-linker(5xGA)-mNeonGreen$_{(5\text{-}177)}$-*K.l. URA3*-mNeonGreen$_{(60\text{-}236)}$-Stop	Amp; *URA3*	This study
pDML222	pFA6a-mNeonGreen$_{(1\text{-}177)}$-*K.l. URA3*-$P_{EF1\alpha}$-mNeonGreen$_{(\text{non-yeast},1\text{-}59)}$-mNeonGreen$_{(60\text{-}236)}$-linker(5xGA)	Amp; *URA3*	This study
pDML223	pFA6a-linker(5xGA)-mNeonGreen$_{(5\text{-}177)}$-mNeonGreen$_{(\text{non-yeast, }178\text{-}236)}$-*K.l. URA3*-mNeonGreen$_{(60\text{-}236)}$-Stop	Amp; *URA3*	This study
pNH603	Single-copy *his3* integration vector with *C.g. HIS3* marker	Amp; *HIS3*	[1]
pNH604	Single-copy *trp1* integration vector with *C.g. TRP1* marker	Amp; *TRP1*	[1]
pUG72	Plasmid with loxP-flanked *K.l. URA3* marker	Amp; *URA3*	[2]
pDH149	pFA6a-AfeI-linker-NmGFPmut3$_{(2\text{-}158)}$-*ura4*-mGFPmut3-BclI	Amp; *ura4*	This study
pDH165	pFA6a-AfeI-linker-NmGFPmut3$_{(2\text{-}158)}$-kanMX6-HSV1tk-mGFPmut3-BclI	Amp; kanMX6 HSV1tk	This study
pDH193	pFA6a-AfeI-linker-NmGFPmut3$_{(2\text{-}158)}$-*ura4*-mGFPmut3-HindIII	Amp; *ura4*	This study

References

1. Chau AH, Walter JM, Gerardin J, Tang C, Lim WA. Designing synthetic regulatory networks capable of self-organizing cell polarization. Cell. 2012;151: 320–332. doi:10.1016/j.cell.2012.08.040

2. Gueldener U, Heinisch J, Koehler GJ, Voss D, Hegemann JH. A second set of loxP marker cassettes for Cre-mediated multiple gene knockouts in budding yeast. Nucleic Acids Res. 2002;30: e23.
