# Supplementary material for: Scarless Gene Tagging with One-Step Transformation and Two-Step Selection in Saccharomyces cerevisiae and Schizosaccharomyces pombe
Source: PLoS One. 2016 Oct 13;11(10):e0163950. doi: 10.1371/journal.pone.0163950 (PMC5063382; doi:10.1371/journal.pone.0163950)
Supplement: S3 Table — (PDF) [file pone.0163950.s012.pdf]

S3 Table. Primers used in this study.

Name	Sequence
DML_P37_R	TAAAACCTAAGAGTCACTTTAAAATTTGTATACAC
DML_P74_F	TCAGTCCTTCTTTAGAAGAGTGAGTGCG
DML_P75_R	CACGCCAAGAAACCCTTGATCC
DML_P110_F	TCGTACGCTGCAGGTCGACGGATCCCCGGGTTAATTAACGGAGCAGGTGCTGGTG
DML_P111_R	ATAAATCATAAGAAATTCGCTTATTTAGAAGTGGCGCGCCTTACTTGTACAATTC
DML_P218_F	TAACGGAGCAGGTGCTGGTGCTGGCGCCGGTGCAGGCGAGGAGGATAACATGGCC
DML_P219_R	AGAAATTCGCTTATTTAGAAGTGGCGCGCCTTACTTGTACAGCTCGTCCATGCCC
DML_P285_F	CCGCTAGGGATAACAGGGTAATATAGATCTGGCTCTATATGTATATAAAGCTGCGGATGC
DML_P286_R	TGATATCATCGATGAATTCGAGCTCGTTTAAACCAAAGCCGAATCCACCACGGTC
DML_P368_F	AATTTGGTTACCACACTGACCTGTCG
DML_P369_R	CGCTTGTCCTTTAAATCACACTTGTTACC
DML_P371_F	AGCGGTACCGGGCCCAAATACCTCGAGACAGTTTATTCCTGGCATCCACTAAATATAATG
DML_P379_F	TAGTTTTGTTAGGCAAGAATTAGCCAAGTC
DML_P380_R	GCGCATTAACAACATGAGATGACAGC
DML_P405_R	GTTTGTTTATGTGTGTTTATTCGAAACTAAGTTC
DML_P442_R	GAGCTCCACCGCGGTGGCGGCCGCTTACTTGTACAATTCGTCCATACCCATAACG
DML_P444_F	CACACATAAACAAACATCGATTAACAAAATGGTTTCTAAGGGTGAAGAAGACAACATGGC
DML_P461_R	ATCCCTGTGTGCTTAGGATCAGATCTATATTACCCTGTTATCCCTAGCGG
DML_P462_F	GATAACAGGGTAATATAGATCTGATCCTAAGCACACAGGGATTGTTGTAC
DML_P463_R	TATCATCGATGAATTCGAGCTCGTTTAAACGCTGATTAGTTATCACATTTTATCCCTATG
DML_P476_R	ACCGTCTGGGTATGGCAAGTATTGG
DML_P499_F	AGCGGTACCGGGCCCAAATACCTCGAGACTATACATAATTTACCCGTTCAGAATGCAATG
DML_P500_R	CCTTAGAAACCATTTTGTTAATCGATATTTGTAATTAAAACTTAGATTAGATTGCTATGC
DML_P504_R	TGTAAAAAAATGCGTTACCACCATCC
DML_P528_R	AAAAGATCACGTGATCTGTTGTATTGGG
DML_P555_F	AAGCTTCGTACGCTGCAGGTCGACGGATCCATGGTTTCTAAGGGTGAAGAAGACAACATG
DML_P556_R	TGTTGTATTGGGATCTCTAGAGTATCTCTTACCGTTACCAGTAGTGTAAGACCAC

DML_P557_F	CTGGTAACGGTAAGAGATACTCTAGAGATCCCAATACAACAGATCACGTG
DML_P558_R	CGGGTAAATTATGTATAGTCCCGGGAGCTCGTTTTATTTAGGTTCTATCGAGGAG
DML_P559_F	CTAAATAAAACGAGCTCCCGGGACTATACATAATTTACCCGTTCAGAATGCAATG
DML_P560_R	CCAAGATCCATGGTTTGTTAATCGATATTTGTAATTAAAACTTAGATTAGATTGC
DML_P561_F	AAGTTTTAATTACAAATATCGATTAACAAACCATGGATCTTGGTTCCACACATCG
DML_P562_R	GGAGACCGGCAGATCCGCGGCCGCATAGGCCACTAGTTGCACCAGCACCAGCACCAGCGCCTGCTCCCTTGTACAATTCGTCCATACCCATAACG
DML_P584_F	CCAATGGCTGCTAACTACTTGAAGAACC
DML_P587_F	GCTGTCAGTTTTTTCCACAGTTTTGG
DML_P588_R	AGCAGTATCTTCATCCAAATGGCCC
DML_P608_F	TCTAAGTTTTAATTACAAATATCGATTAACAAAATGCCATGGATCTTGGTTCCACACATC
DML_P609_R	TTCTTCAAGTAGTTAGCAGCCATTGGC
DML_P619_F	GCTTCGTACGCTGCAGGTCGACGGATCCATGGTTTCTAAGGGTGAAGAAAACAACATGGC
DML_P620_R	AGATCACGTGATCTGTTGTATTGGGATCTCTAGAGTGACCACCGTCCTTCAACTTCAATC
DML_P621_F	ATCTAAGTTTTAATTACAAATATCGATTAACAAAATGCCATTCGCTTGGGACATCTTGTC
DML_P622_R	CGGCAGATCCGCGGCCGCATAGGCCACTAGTTGCACCAGCACCAGCACCAGCGCCTGCTCCCTTGTACAATTCGTCCATACCACCAGTAG
DML_P626_F	TCGTACGCTGCAGGTCGACGGATCCGGCTCCGGTTCTAAGGGTGAAGAAGACAACATGGC
DML_P630_R	GGGAGACCGGCAGATCCGCGGCCGCATAGGCCACTAGTGCCGGAACCCTTGTACAATTCGTCCATACCCATAACG
DML_P642_R	TGTGTGGAACCAAGATCCATGGCCCGGGAGCTCGTTTTATTTAGG
DML_P643_F	TAAATAAAACGAGCTCCCGGGCCATGGATCTTGGTTCCACACATC
DML_P712_F	CGAAGAATTGAACTTGAAGTCTACTAAGGG
DML_P713_R	GCAGATCCGCGGCCGCATAGGCCACTAGTTTACTTGTACAATTCGTCCATACCCATAACG
DML_P738_F	CTAATCTAAGTTTTAATTACAAATATCGATTAACAAAATGGTGAGCAAGGGCGAGGAGGATAACATGGCC
DML_P739_R	TGTGGAACCAAGATCCATGGGGGAGAACTGGAGGTCACCCTTG
DML_P740_F	GGGTGACCTCCAGTTCTCCCCCATGGATCTTGGTTCCACACATC
DML_P742_F	TACTGGTAACGGTAAGAGATACCGGAGCACTGCGCGGACC
DML_P743_R	GATCTGTTGTATTGGGATCTCTAGATTATTTATATAGCTCGTCCATGCCCATCACATC
DML_P744_R	TGATGGCTGTACCGATAGGAACGC
tdh1_C_F1	TTTATCGATCTTGAATACATGGCCTACATGTT
tdh1_C_R1	TCCACCTCCACCACTAGCACTACCAGCGTTGTCCTTGGCGGCAGTGTAG

tdh1_C_F2	TACACATGGCATGGATGAACTATACAAATAACTAGCTAATCATCCCGATGCTAAGA
tdh1_C_R2	AGATTGACAATAAACATTTTCGTTTTTGTTGATTAACATG
